# Veterinary Drug Prescribing Practices at Selected District Veterinary Clinics of Rift Valley Areas of Ethiopia

**DOI:** 10.1155/2021/6669036

**Published:** 2021-11-08

**Authors:** Monenus Etefa, Ashenafi Feyisa Beyi, Dinka Ayana, Tariku Jibat Beyene, Takele Beyene Tufa

**Affiliations:** ^1^Jimma University, College of Agriculture and Veterinary Medicine, Jimma, Ethiopia; ^2^Veterinary Microbiology and Preventive Medicine, Iowa State University, Ames, IA 50011, USA; ^3^College of Veterinary Medicine and Agriculture, Addis Ababa University, Bishoftu, Ethiopia; ^4^Department of Preventive Veterinary Medicine, The Ohio State University, Columbus, OH 43210, USA; ^5^Department of Farm Animal Health, Faculty of Veterinary Medicine, Utrecht University, Utrecht, Netherlands

## Abstract

The rational use of drugs in veterinary medicine has various significances, such as reducing the risk of drug resistance, increasing efficacy, reducing drug residue, and decreasing adverse drug reactions. A retrospective study was conducted to assess veterinary drug prescribing practices at Batu and Arsi-Negelle district veterinary clinics in the rift valley areas of Ethiopia. A total of 2,464 cases were recorded from the case registration books at both the clinics for diseases treated between September 2012 and February 2015. The study results showed that for a total of 2,464 cases diagnosed at both clinics, 3,811 different drugs were prescribed, with an average per encounter of 1.6. Among the total drugs, oxytetracycline, ivermectin, penstrep, sulfa drugs, and albendazole were the most leading prescribed drugs with a frequency of 43.0%, 17.6%, 10.2%, 6.5%, and 1.3%, respectively. All drugs were prescribed by the generic name without any laboratory support of the disease. About 68.3% of the cases were diagnosed by unspecified professionals, whereas 21.7% and 10.1% were done by animal health assistants and veterinarians, respectively. The prescribing practices showed 61.0% of antibiotics and 29.7% of anthelmintics where 45.3% and 54.7% of antibiotics and 17.8% and 82.2% of anthelmintics were given at Batu and Arsi-Negelle veterinary clinics, respectively. Of the prescribed drugs, 4.6% oxytetracycline and 2.6% penstrep were prescribed irrationally to treat diseases that were tentatively diagnosed as parasitic cases. Similarly, 40.5% ivermectin and 17.7% albendazole were prescribed for bacterial infections. In conclusion, this study revealed problems in antibiotics and anthelmintics use, description of routes of administration and length of treatment, and shortage of laboratory diagnostic facilities. Therefore, veterinary drugs, particularly antibiotics and anthelmintics, should be used appropriately to safeguard the public from residual drug impacts and resistance development.

## 1. Introduction

The rational use of drugs is the use of the right medicines, correct dosage, and correct cost, which is well reflected in the World Health Organization (WHO) definition: “Rational use of drugs requires that patients receive medications appropriate to their clinical needs, in doses that meet their requirements for an adequate period, at the lowest cost” [1]. However, the irrational use of medicines is when one or more of these conditions are not met, for example, too many medicines are prescribed per patient, injections are used where oral formulations would be more appropriate, antimicrobial agents are prescribed in inadequate doses or duration, or antibiotics prescribed for nonbacterial infections, thereby contributing to the growing problem of antimicrobial resistance and prescriptions do not follow clinical guidelines [2].

Problems like lack of information, poor communication between health professionals and animal owners, lack of diagnostic facilities, demand from the owners, and high burden of diseases with overlapping clinical symptoms (e.g., pain, fever, and depression are common symptoms for different conditions which require different drugs) [3] lead to the irrational use of drugs causing ineffective and unsafe treatment, exacerbation or prolongation of illness, distress and harm to the patient, and increase the cost of treatment [4, 5].

Antibiotics are widely used in healthy food-producing animals to promote growth and prevent diseases. This practice favors the emergence and spread of resistant bacteria in both animal and human populations. The routine use of antimicrobials in vast numbers of healthy animals is likely to result in the emergence and spread of antimicrobial-resistant bacteria and cause resistant infections in animals and humans. Likewise, anthelmintic resistance is becoming an increasing global problem resulting from the misuse of these drugs. Resistance to anthelmintic by ruminant nematode parasites is a growing problem throughout the world [6]. Food animals and foods of animal origin are traded worldwide; thus, drug resistance affecting the food supply of one country becomes a potential problem for other countries [2].

There are essential measures to be taken to improve rational drug prescribing. These are critical assessment and evaluation of benefits and risk of drug used; safety and cost of the drug with the existing drug for some indication; identification of the patient's problem based on symptoms and recognizing the need for action; diagnosis of the disease, identifying underlying cause, and motivating factors that may be specific as in infectious disease or nonspecific; use of possible intervention or treatment which may be nondrug treatment or drug treatment by choosing from different alternatives based on efficacy, convenience, and safety of drugs including drug interactions and high-risk group of patients; start the treatment by writing an accurate and complete prescription, for example, the name of drugs with dosage forms and schedule and total duration of the treatment [7–10].

Currently, in the clinical practice of human and veterinary medicine throughout the world, large amounts of antibiotics are used. Equally, many scientists intensively work on the discovery and synthesizing of new drugs with a broader antimicrobial spectrum, stronger action, and a more satisfactory safety profile. Most failures during antimicrobial therapy may occur when the pathogenic microorganism is unknown and a combination of two or more drugs administered empirically. To avoid these mistakes, clinically confirmed, effective antimicrobial combinations should be used [11]. Globally, more than half of all medicines are prescribed, dispensed, or sold improperly, and 50% of the human patients fail to take them correctly. This is more wasteful, expensive, and dangerous, both to the health of the individual animal patient and to the public as a whole that magnifies the problem of misuse of antimicrobial agents [2].

In humans, assessments of drug use patterns with the WHO drug use indicators are becoming increasingly necessary to promote rational drug use. They are now widely accepted as a global standard for problem identification and used in developing countries [12, 13]. In Ethiopia, a survey conducted on human subjects at hospitals located in different regions of the country revealed irrational drug use [14, 15]. However, in veterinary practice, a few published reports on the rational use of veterinary drugs in the country in general, although different studies were conducted by Beyene et al., revealed irrational use of drugs in veterinary clinics [16].

Hence, the present study was designed to evaluate the rational use of veterinary drugs and to compare magnitudes of different drugs commonly used for the treatment of food animal diseases in the Batu and Arsi-Negelle district veterinary clinics in general and to describe current treatment practices and to evaluate the adherence of the prescriber to the national veterinary treatment guidelines.

## 2. Materials and Methods

### 2.1. Study Area and Period

The study was conducted from November 2014 to March 2015 at Batu veterinary clinic, located in the East Shoa zone of the Oromia regional state, and Arsi-Negelle veterinary clinic, located in the Western Arsi zone of the Oromia regional state. Batu has a latitude of 7°56′N and a longitude of 38°43′E with an elevation of 1,643 meters above sea level. The average annual temperature in Batu is 19.3°C and 837 mm of precipitation falls annually. Arsi-Negelle is found in the Western Arsi zone of Oromia having a latitude and a longitude of 7°21′N and 38°42′E, respectively, with an altitude of 2,043 meters above sea level and annual rainfall and temperature of 1,300 mm and 21.5°C, respectively [17].

### 2.2. Study Design

A retrospective and cross-sectional study was designed to assess rational drug use and to compare commonly used drugs for the treatment of animal diseases at Batu and Arsi-Negelle district veterinary clinics based on WHO drug use indicators as described in [1]. The sample was selected using a systematic random sampling method, and the sampling unit was an animal patient encountered at both veterinary clinics for the treatment of acute and/or chronic illness. Secondary data are the source of information. Accordingly, data were collected from the case book records from the office of both clinics by using systematic random sampling in which every third case and tenth case were recorded at Batu and Arsi-Negelle veterinary clinics, respectively.

### 2.3. Study Population

The study was conducted between November 2014 and March 2015 on food animal patients (cattle, sheep, goats, and chicken of all ages and sex groups) that were admitted to Batu and Arsi-Negelle district open-air veterinary clinics and treated with drugs. All other nonfood animals (e.g., pets and equines) were excluded from the study. A total of 2,464 case data were collected to evaluate the rational use of veterinary drugs both at Batu and Arsi-Negelle veterinary clinics.

### 2.4. Data Collection

Data were collected in data collection format retrospectively using case registration books at both the clinics, namely, Batu and Arsi-Negelle veterinary clinics. The specific types of data necessary to measure the prescribing indicators were recorded for each animal patient encounter and entered directly into an ordinary prescribing indicator form. For this particular study, 2464 prescriptions that contain the animal's characteristics (age, sex, breed, body condition, clinical signs, and symptoms observed); disease diagnosis (name, empiric or physical clinical examination, and confirmatory laboratory tests used); prescribed drugs (type, naming (generic or brand), number of drugs prescribed, route of administration, duration of treatment); and prescriber's signature, level of education, and years of experiences were collected retrospectively from about 16500 prescriptions written for two and half years period from September 2012 to February 2015. Accordingly, the evaluation of the rational use of veterinary drugs was made based on generic prescription and antimicrobials and anthelmintics prescribed for tentatively diagnosed clinical cases.

### 2.5. Data Analysis

All data in the ordinary prescribed indicator recording form were entered into a Microsoft Excel spreadsheet (version 2010) and imported and analyzed using SPSS (Version 20). Descriptive statistics such as frequencies, percentages, and cross-tabulation were used to describe the characteristics of the drugs. The Chi-square test was used to compare categorical variables where appropriate. All statistical tests were two-sided, and *p* ≤ 0.05 was considered significant.

### 2.6. Prescribing Indicators

There was no available guideline for prescribing indicators used in veterinary medicine. As a result, the PRESCRIBING indicators were used in this study [18]. The indicators were pretested and slightly modified to match with clinical practice in veterinary medicine so that they could be used easily to provide accurate data. The final versions of the pretested indicators are as follows:

1. The average number of drugs prescribed per encounter was calculated by dividing the total number of different drug products prescribed with the number of encounters surveyed to measure the degree of polypharmacy.
2. The percentage of drugs prescribed by the generic name was calculated by dividing the number of drugs prescribed by the generic name with the total number of drugs prescribed, multiplied by 100 to measure the tendency of prescribing by generic name.
3. The percentage of encounters in which antimicrobials, anthelmintics, and other drugs prescribed were calculated by dividing the number of patient encounters in which the drug was prescribed with the total number of drugs prescribed and multiplied by 100 to measure the overall use of commonly overused (irrationally prescribed) and costly forms of drug therapy.
4. The percentage of drugs prescribed for each disease encountered was calculated by dividing the number of drugs prescribed for each disease for the total number of encounters and multiplied by 100.

## 3. Results

A total of 2,464 presented cases to clinics were assessed from Batu (1163) and Arsi-Negelle (1301) district veterinary clinics. A retrospective study has shown that 3,811 drugs were prescribed, and the average number of drugs per encounter was 1.6 with a maximum of 3 and a minimum of 1. Among 3,811 prescribed drugs, 1,637 (43.0%) Oxytetracycline, 671 (17.6%) Ivermectin, and 389 (10.2%) Penstrep were most commonly used. Of these drugs, 45.0% and 55.0% of Oxytetracycline, 16.5% and 83.5% of Ivermectin, and 48.1% and 52.9% of Penstrep were prescribed in Batu and Arsi-Negelle, respectively (Table 1).

The rational drug use evaluation showed that antimicrobials, anthelmintics, antimicrobial with anthelmintic combinations, antimicrobial, and/or anthelmintic with other drugs combinations were prescribed (Table 2). Accordingly, 61.0% and 12.2% and 27.9% and 22.4% of individual and combined antibiotics and individual anthelmintics and its combination with antibiotics were prescribed, respectively. However, most of the combinations were done at the Arsi-Negelle clinics than at the Batu clinics except for the combination of the antibiotics, which was combined at Batu (64.0%) than Arsi-Negelle (36.0%).

Among the total 2,464 patient encounters, the relationship between tentatively diagnosed diseases and drugs prescribed was evaluated. The results showed that Oxytetracycline was prescribed for 737 (45.0%) treatments of bacterial infection, 108 (6.6%) pneumonia cases, 76 (4.6%) gastrointestinal parasites, 81 (5.0%) pasteurellosis cases, and 68 (4.2%) grain overload cases. Likewise, 272 (40.5%), 38 (5.7%), 58 (8.6%), 21 (3.1%), 19 (2.8%), 69 (10.3%), 7 (0.9%), and 18 (2.7%) times Ivermectin was prescribed against bacterial infection, pneumonia, gastrointestinal parasites, pasteurellosis, grain overload, enteritis, mastitis, and bloat, respectively. Albendazole was also prescribed for the treatment of 9 (17.7%) bacterial infection cases (Table 3).

All the drugs prescribed between the study periods were by generic naming. However, most of the drug prescriber's status was not identified at Batu and unidentified at all at the Arsi-Negelle veterinary clinic. Although almost all the drugs were prescribed for EVDL, the level of irrational use of drugs was still remaining higher (Table 4), that is, 2648 (69.5%) drugs were prescribed irrationally only from the perspectives of clinical signs observed and drugs administered with a higher percentage at Arsi-Negelle (59.2%) than Batu (40.8%) veterinary clinic centers.

## 4. Discussion

Retrospective and cross-sectional study results of drugs used at both clinics of the study sites indicated that a total of 2,464 animals were presented to the clinics and 3,811 drugs were prescribed with an average per case of 1.6 (with the maximum of 3 and a minimum of 1 drug) which is almost equivalent to the WHO standard prescription guidelines that range between 1.6 and 1.8 [19]. Although the average of the total prescribed drugs is similar to the WHO standard prescription guidelines, there is variation between the study sites, that is, below the standard for Batu (1.03) but exceeds the standard of Arsi-Negelle (2.01). There is no published study on veterinary drug prescription patterns in Ethiopia. However, some studies performed on human subjects at Jimma Hospital, Gondar, Bahir Dar, and Debra Tabor revealed that the average number of drugs per encounter was 1.59, 0.98, 1.8, and 2.2, respectively [20, 21]. Likewise, research conducted in various countries like India, Nepal, Niger, Nigeria, Cameroun, Morocco, Mozambique, and Uganda indicated that there is a higher average percentage of drugs prescribed per encounter ranging between 2.2 and 5.3 that probably facilitates adverse drug reactions and consequently not achieving desired therapeutic outcomes [22–25]. A high average number of drug prescriptions might be due to a lack of therapeutic training of prescribers or a shortage of therapeutically correct drugs. However, the low values might show inadequate availability of drugs [18].

The percentage of encounters by which antibiotics were prescribed at Batu and Arsi-Negelle veterinary clinics were 45.3% and 54.7% with a total average of 61.0%, and that of anthelmintics was 19.9% and 80.1% with a total average of 27.9%, respectively (Table 2). The ideal standard percentage of encounters in which antibiotics were prescribed for humans is less than 30 for antibiotics [12, 19]. The percentage of antimicrobials administered at these study sites is higher when compared with the results from other studies of human cases. For instance, the findings of the research conducted in Malawi (34%), Indonesia (43.1%), Bangladesh (25%), and Tanzania (39%) [26, 27] resulted in lower values than the current results. However, reports of [28] (72.8%) are found to be higher than these and current study results. This finding suggests that antimicrobials and anthelmintics are overprescribed and need to be regulated. The high percentage of antibiotics prescribed in this study setting may be due to inaccuracy in the disease, unavailability of diagnostic aids for confirmatory tests, absence of the right drug, prescribers' low belief of the therapeutic efficacy of the antibiotics, and prescribers' knowledge [29].

A national baseline study on drug use indicators (human subjects) in Ethiopia in 2002 also showed that the percentage of encounters in which an antibiotic was prescribed be 58.1% [15], which was nearly equivalent to the current result. Even although clinical indications were one of the diagnostic approaches, febrile conditions (263 (36.0%)) were treated irrationally by anthelmintic. Therefore, this finding indicates that there is overprescribing of antibiotics when compared to reports from other studies and WHO recommendation standard of less than 30% [3], which may result in unwanted consequences. Like antibiotics, there were also reports on anthelmintic resistance because of irrational anthelmintic prescription. For instance, resistance to Albendazole and Ivermectin by Cooperia and *Haemonchus* species were reported in several countries [30, 31].

The basic purpose of veterinary drugs is to protect the health and welfare of animals [31, 32]. However, 101 (4.35%) antimicrobials (76 Oxytetracyclines, 10 Penstrep, 10 sulfa drugs, and 5 penicillins) (*p* < 0.005) were prescribed irrationally to treat diseases that were tentatively diagnosed as parasitic cases. The association between antibiotic used and parasitic cases is statistically significant (*p* < 0.001). Such a high level of antibiotic prescription may be accounted for by the assumption that every medical condition will very likely present with a bacterial complication. In addition, 272 (40.5%) Ivermectin (*p* > 0.005) and 9 (17.65%) Albendazole (*p* < 0.005) where prescribed irrationally for cases diagnosed as bacterial infection (Table 3).

Even though all of the cases encountered in the Batu and Arsi-Negelle veterinary clinics received drug therapy after they had been tentatively diagnosed, the route of drug administration and length of treatment (particularly at Batu) were not indicated for all the prescribed drugs, which revealed the presence of irrational drug use. Such practice of drug prescription (prescription of unnecessary drugs, inappropriate choice of route, dose, and duration of antibiotics [29]) in food-producing animals probably results in drug residues, which may promote allergenic, toxic, mutagenic, teratogenic, or carcinogenic, and it may favor the emergence of resistant microbial strains within a host [32].

In other ways, from the total of drugs prescribed (3811) at these study sites, oxytetracycline (1637 (43.0%)) was the most commonly used even though resistance against this drug, [33] followed by 671 (16.6%) ivermectin, 389 (10.2%) penstrep, 247 (6.5%) sulfa drugs, 51 (1.3%) albendazole, and 816 (21.4%) are other different drugs (multivitamin, penicillin, copper sulfate, Tricalbendazole, tetraconazole, anesthetic agents, diminazene aceturate, iodine, intramammary infusion, digestion powder, vitamin K, and calcium borogluconate). From these prescribed drugs, 736 (45.0%) and 901 (55.0%) oxytetracycline, 111 (16.54%) and 560 (83.46%) ivermectin, 187 (48.07%) and 202 (51.93%) penstrep, 100 (40.5%) and 147 (59.5%) sulfa drugs, and 6 (11.76%) and 45 (88.24%) albendazole prescriptions were done at Batu and Arsi-Negelle veterinary clinics, respectively (Table 1). From these results, most of the drug prescriptions (particularly albendazole, Ivermectin, sulfa drugs, and oxytetracycline), were done at Arsi-Negelle than Batu, whereas the percentage of Penstrep use was almost equal.

All drugs (100%) were prescribed by the generic name (Table 2). This is the highest number even when compared with drugs prescribed for human patients in different countries such as Bangladesh (65%) and Nigeria (63.8%) ([34, 35]. The high value of the average percentage of drugs prescribed by a generic name showed that the veterinary professionals working at clinics are conversant with the standard practice of prescribing using generic names. Increasing generic prescribing has been proven to rationalize the use as well as reduce the cost of drugs to patients [36]. On the other hand, the length of treatment of prescribed drugs was not totally indicated for Batu, whereas drugs are administered for stat, once every day for three days, once per day for four days, and once per day for five days at the Arsi-Negelle district veterinary clinics.

Drug prescribers and their work experience were not specified at all at Arsi-Negelle unlike at Batu, in which 534 (21.7%) cases were treated by animal health assistance (having work experience of greater than seven years) and 248 (10.1%) veterinarians (having work experiences of less than seven years), but 381 (22.7%) were prescribed by unidentified personnel. This may also be able to stimulate adverse drug effects because of irrational use.

Even though combination drugs are irrational because of their doubtful stability, reducing the efficacy increases the risk of side effects and may also needlessly increase the cost; 11.9% of antibiotics, 22.0% of antibiotics and anthelmintics, and combination were prescribed, whereas a combination of different drugs like antibiotics, anthelmintics, and vitamins account 9.7% (Table 3). Most of these combinations were done at the Arsi-Negelle than at the Batu district veterinary clinic except antibiotics combination which was combined at the Batu clinics (64.0%) than at Arsi-Negelle clinics (36.0%). Combination drugs should be only used when there is no alternative of single drugs available for treatment or no alternative single drug is cost-effective for the purpose. One of the main causes of irrational use of medicines may be the availability of irrational fixed-dose combinations [37].

## 5. Conclusions

The pattern of rational drug use at the current study clinics was generally not satisfactory, although the level of generic prescription was recommendable compared to the WHO standards. This study showed that the use of antibiotics and anthelmintics are too high and there may therefore be the need to establish protocols on drug prescription. The overall picture of drug use suggests that the indicators at these facilities are not yet at the optimal level and need some interventions. The result obtained in this study provides a baseline for veterinarians and concerned bodies of the respective districts of the study sites in particular and the country, in general, to monitor and make the necessary educational and managerial interventions to improve the situation in veterinary drugs use. Hence, we suggest corrective measures should be undertaken to facilitate rational drug use in food-producing animals through improving the availability of diagnostic facilities at veterinary clinics to improve patient misdiagnoses and awareness of veterinary clinical practitioners about rational drug use. Further research should also be conducted to evaluate the rational veterinary drug use at different agro-ecologies of the country to take appropriate in general and food animals in particular.

## Figures and Tables

**Table 1:** Commonly prescribed drugs at Batu and Arsi-Negelle district veterinary clinics between the period of September 2012 to February 2015.

Commonly prescribed drugs	Total N (%)	Name of clinics	
		Batu N (%)	Arsi-Negelle N (%)
Total drugs	3811	1199 (31.5%)	2612 (68.5%)
Oxytetracycline	1637 (43.0%)	736 (45.0%)	901 (55.0%)
Ivermectin	671 (17.6%)	111 (16.5%)	560 (83.5%)
Penstrep	389 (10.2%)	187 (48.1%)	202 (52.9%)
Sulfa drug	247 (6.5%)	100 (40.5%)	147 (59.51%)
Albendazole	51 (1.3%)	6 (11.8%)	45 (88.2%)
Others*∗*	816 (21.4%)	59 (7.2%)	757 (92.8%)

*N* = frequency; others*∗* = penicillin, tricalbendazole, multivitamin, tetraconazole, copper sulfate, anesthetic agents, diminazene aceturate, intramammary infusion, digestion powder, vitamin K, and calcium borogluconate.

**Table 2:** Association between prescribed drugs and their combinations across the respective vet clinics.

Variables		N (%) from total	Batu N (%)	Arsi-Negelle N (%)
Antibiotic	No	1487 (39.0%)	111 (79.3%)	29 (20.7%)
	Yes	2324 (61.0%)	1052 (45.3%)	1272 (54.7%)
Anthelmintics	No	2748 (72.1%)	1638 (59.6%)	1110 (40.4%)
	Yes	1063 (27.9%)	212 (19.9%)	851 (80.1%)
Drug combination	AB + AB	465 (12.2%)	298 (64.0%)	167 (36.0%)
	AB + AH	854 (22.4%)	35 (4.1%)	819 (95.9%)
	AH + AH	35 (0.9%)	0 (0.0%)	35 (100.0%)
	Others	572 (15.0%)	67 (11.7%)	505 (88.3%)
	Single	1885 (49.5%)	799 (42.4%)	1086 (57.6%)
Generic prescription		3811 (100.0%)	1199 (31.5%)	2612 (68.5.0%)
Drug prescribed from EVDL		3811 (100.0%)	1199 (31.5%)	2612 (68.5%)

AB + AB = antibiotics combination, AB + AH = antibiotics and anthelmintics combination, AH + AH = anthelmintics combinations, EVDL = essential veterinary drug list, and *N* = frequency.

**Table 3:** Association between diseases and drugs administered at Batu and Arsi-Negelle district veterinary clinics.

Diseases diagnosed	N (%)	Drugs administered				
		Oxytetracycline	Ivermectin	Penstrep	Sulfa drug	Albendazole
BI	941 (38.2%)	737 (45.0%)	272 (40.5%)	103 (26.6%)	59 (23.9%)	9 (17.7%)
Pneumonia	197 (8.0%)	108 (6.6%)	38 (5.7%)	64 (16.5%)	10 (4.0%)	2 (3.9%)
GIP	128 (5.2%)	76 (4.6%)	58 (8.6%)	10 (2.6%)	10 (4.05%)	18 (35.3)%
Pasteurellosis	123 (5.0%)	81 (5.0%)	21 (3.1%)	23 (5.9%)	9 (3.6%)	1 (2.0%)
CHO overload	106 (4.3%)	68 (4.2%)	19 (2.8%)	14 (3.6%)	11 (4.5%)	0 (0.0)
Enteritis	92 (3.7%)	21 (1.3%)	69 (10.3%)	69 (17.7%)	69 (27.9)	2 (3.9)
Mastitis	89 (3.6%)	45 (2.75%)	7 (0.9%)	1 (0.26%)	1 (0.4%)	1 (2.0%)
Bloat	59 (2.4%)	51 (3.1%)	18 (2.7%)	3 (0.77%)	3 (1.2%)	0 (0.0)
Others	729 (29.6%)	450 (27.5%)	169 (25.2%)	102 (26.2%)	75 (30.4%)	18 (35.3%)

BI = bacterial infection; GIP = gastrointestinal parasites.

**Table 4:** Relationship between prescriber's status and drugs used across the two clinics.

Variables	Prescribers status	N (%)	Batu N (%)	Arsi-Negelle N (%)
Educational level	Unknown	1682 (68.3%)	381 (22.7%)	1,301 (77.3%)
	Vet	248 (10.1%)	248 (100%)	0.0 (0.0%)
	AHA	534 (21.7%)	533 (100%)	0.0 (0.0%)
Work experience	Unknown	1682 (68.3%)	381 (22.7%)	1,301 (77.3%)
	<7 yrs	248 (10.1%)	248 (100%)	0.0 (0.0%)
	>7 yrs	534 (21.7%)	533 (100%)	0.0 (0.0%)
Average of rational*∗*		1163 (30.5%)	689 (59.2%)	474 (40.8%)
Average of irrational*∗*		2648 (69.5%)	1081 (40.8%)	1567 (59.2%)

*N* = frequency; AHA = animal health assistant; vet = veterinarian; EVDL = essential veterinary drug list; *∗* = determined based on clinical signs and drugs administered; unknown = not possible to identify.

## Data Availability

The datasets used and/or analyzed during the current study are available from the corresponding author on reasonable request.

## Abbreviations

CSA: Central Statistical Agency
EPA: Ethiopian Pharmaceutical Association
EVDL: Essential veterinary drug list
INRUD: International Network for the Rational Use of Drugs
SPSS: Statistical Package for Social Sciences
WHO: World Health Organization.
